# Low CO_2_ Sensitivity of Microzooplankton Communities in the Gullmar Fjord, Skagerrak: Evidence from a Long-Term Mesocosm Study

**DOI:** 10.1371/journal.pone.0165800

**Published:** 2016-11-28

**Authors:** Henriette G. Horn, Nils Sander, Annegret Stuhr, María Algueró-Muñiz, Lennart T. Bach, Martin G. J. Löder, Maarten Boersma, Ulf Riebesell, Nicole Aberle

**Affiliations:** 1 Alfred-Wegener-Institut Helmholtz-Zentrum für Polar- und Meeresforschung, Biologische Anstalt Helgoland, Helgoland, Germany; 2 GEOMAR Helmholtz Centre for Ocean Research Kiel, Kiel, Germany; 3 Animal Ecology I, University of Bayreuth, Bayreuth, Germany; 4 University of Bremen, Bremen, Germany; University of Hyogo, JAPAN

## Abstract

Ocean acidification is considered as a crucial stressor for marine communities. In this study, we tested the effects of the IPCC RPC6.0 end-of-century acidification scenario on a natural plankton community in the Gullmar Fjord, Sweden, during a long-term mesocosm experiment from a spring bloom to a mid-summer situation. The focus of this study was on microzooplankton and its interactions with phytoplankton and mesozooplankton. The microzooplankton community was dominated by ciliates, especially small *Strombidium* sp., with the exception of the last days when heterotrophic dinoflagellates increased in abundance. We did not observe any effects of high CO_2_ on the community composition and diversity of microzooplankton. While ciliate abundance, biomass and growth rate were not affected by elevated CO_2_, we observed a positive effect of elevated CO_2_ on dinoflagellate abundances. Additionally, growth rates of dinoflagellates were significantly higher in the high CO_2_ treatments. Given the higher Chlorophyll *a* content measured under high CO_2_, our results point at mainly indirect effects of CO_2_ on microzooplankton caused by changes in phytoplankton standing stocks, in this case most likely an increase in small-sized phytoplankton of <8 μm. Overall, the results from the present study covering the most important part of the growing season indicate that coastal microzooplankton communities are rather robust towards realistic acidification scenarios.

## 1. Introduction

Atmospheric CO_2_ concentrations have increased considerably from 280 μatm at pre-industrial times to currently about 400 μatm [1] and are predicted to reach up to 1000 μatm by the end of this century (IPCC scenario RPC6.0) [2]. The oceans act as a major CO_2_ sink and have absorbed about 30% of the anthropogenic CO_2_ since the beginning of the industrial revolution [3]. This obviously has affected the ocean’s carbonate system, leading to increased CO_2_ and bicarbonate (HCO_3_^-^) concentrations as well as a decrease in carbonate ion concentrations (CO_3_^2-^) and pH. This drop in pH is referred to as ocean acidification (OA). While there are differences in CO_2_ uptake depending on the region [3], an overall pH decrease of about 0.3 units is expected until the end of the 21^st^ century [4].

Microzooplankton (MZP), plankton within the size class from 20 to 200 μm, is a heterogeneous group consisting of heterotrophic and mixotrophic protists as well as micrometazoa. Often grazing on an average 60–75% of the daily primary production, it is a dietary competitor for larger mesozooplankton [5, 6]. Accordingly, MZP can have a strong impact on biomass and species composition of the phytoplankton community and even play an important role in suppressing phytoplankton blooms, especially at times when mesozooplankton grazing is low [7]. Moreover, MZP can also be strongly top-down controlled itself, as it is a preferred food source for mesozooplankton such as copepods [5, 8].

OA is predicted to affect different marine biological and biogeochemical processes, potentially resulting in adverse effects not only on the species level but also on the community and ecosystem level [9]. Potential direct effects of OA on MZP have been identified as e.g. changes in intracellular pH or enzyme activities [10], and indeed negative effects such as a decrease in biomass or the inhibition of growth have been reported for some species of MZP [11, 12]. Others, however, were not directly affected by a change in pH [13]. Nonetheless, CO_2_ effects can also be transmitted indirectly, via changes in phytoplankton availability, community composition, or food quality [14, 15]. Based on the enhanced growth of especially small-sized phytoplankton species which benefit from the higher carbon availability under OA [16–19], OA has the potential to lead to an increase in MZP productivity as well.

Despite of the pivotal role of MZP, small-scale laboratory experiments providing information about the impacts of OA on single species or simplified food webs are comparatively rare in contrast to the number of studies available for phytoplankton or mesozooplankton (e.g. [20, 21]). Additionally, there is a lack of information about OA impacts on community level. Mesocosm studies are useful to fill this gap as they allow us to gain insight into the effects of OA on the plankton community and whether biotic interactions could dampen or amplify known responses in MZP [15, 22]. The studies available so far indicate that MZP communities, especially in coastal areas, are rather tolerant to OA at incubation times up to weeks [22–25]. Yet, to study evolutionary adaptations to OA which are likely to occur due to the short generation times of planktonic organisms, long-term experiments would be required [26]. Experiments with longer durations are also necessary to allow observing possible numerical responses of mesozooplankton as it reacts time-delayed to CO_2_-induced changes on phytoplankton or MZP level. Consequently, an in-situ mesocosm approach using a natural plankton assemblage and a sufficiently large incubation volume to allow for a long self-sustained runtime is a step towards the understanding of effects on ecosystem level [27].

We investigated the impacts of high CO_2_ levels on natural plankton communities during a long-term mesocosm study in the Gullmar Fjord, Skagerrak. Starting before the onset of the spring bloom in March, the long runtime until end of June made it possible to follow the natural succession of a plankton community during the transition from spring to summer. In the following, we will present the analysis of MZP succession patterns focusing on ciliates and heterotrophic dinoflagellates and their interactions with phytoplankton.

Moreover, grazing experiments should provide additional information both regarding the grazing impact of MZP and indirect effects of a high CO_2_ level which are more likely to be detected when MZP is released from grazing pressure. Our hypotheses considering the effects of high CO_2_ on MZP were as follows:

1. Elevated CO_2_ will not directly affect MZP communities due to their high CO_2_ tolerance.
2. An increase in phytoplankton biomass at high CO_2_ conditions (due to positive effects on photosynthesis) will lead to enhanced MZP biomass and grazing rates.
3. Small sized phytoplankton will profit from high CO_2_ levels which is in favor of MZP grazers but not mesozooplankton thus in turn, grazing pressure on MZP will increase.

## 2. Material and methods

### 2.1 Experimental design

The setup is described in detail by Bach et al. [28], including mesocosm design, CO_2_ addition, and maintenance work during the experiment, to which we refer the reader for further information. In short, ten”Kiel offshore mesocosms for future ocean simulations” (KOSMOS) [27] with a volume of 55 m^3^ each were moored in the Gullmar Fjord on the Swedish west coast at 58°15’9 N, 11°28’7 E in January 2013. Each mesocosm made of polyurethane foil had a diameter of 2 m and was 19 m long, with a conical sediment trap at the lower end. The upper part of the mesocosms was ~2 m above the sea surface, open to allow gas exchange with the surrounding and protected against birds with a transparent roof mounted on the floatation frame. The experiment ran from 7 March till 26 June 2013. Upon closing of the mesocosms, a net with 1 mm mesh size was passed through the enclosed pre-bloom seawater to remove large organisms. Five mesocosms served as control with ambient CO_2_ levels while CO_2_-enriched seawater was added to the other five.

The chosen CO_2_ level of 760 μatm corresponds to the conditions expected for the end of the 21^st^ century according to IPCC scenario RPC6.0 [2]. In order to compensate for outgassing, CO_2_-enriched water was added to the high CO_2_ treatments at five time points (days 17, 46, 48, 68 and 88). Herring larvae (*Clupea harengus*) and sea urchin larvae (*Strongylocentrotus droebrachiensis*) were added to the mesocosms on day 48 and day 56 of the experiment, respectively. Regular sampling every 2^nd^ day included CTD casts, water column sampling, and sediment sampling. Plankton net hauls were performed every eight days. For the water samples, two 10 L carboys per mesocosm were filled using an integrating water sampler (IWS III, Hydro-Bios) over a depth of 0–17 m. Plankton samples were taken from the carboys as soon as they were back on shore.

### 2.1 Sampling and identification of MZP

MZP samples were taken once per week. 250 mL of mesocosm water was transferred into brown glass bottles and fixed with acidic Lugol’s solution (1% final concentration). MZP was counted and identified with an inverted microscope (Zeiss Axiovert 135) using the Utermöhl method [29]. Depending on the plankton abundance, 50 or 100 mL of each sample were transferred into a sedimentation chamber. After 24 h of sedimentation, the whole surface of the chamber was counted at 200-fold magnification to reduce the counting bias against rare species and to assure comparability of the counts both at high and low abundances.

MZP was identified to the lowest possible taxonomic level (species or genus level) according to Carey [30], Montagnes et al. [31], and Kraberg et al. [32] and otherwise grouped into size classes. Most dinoflagellates are capable of heterotrophic feeding modes and can be considered as mixotrophic MZP. Only few taxa such as *Ceratium* sp. were considered as predominantly autotrophic and thus included in the phytoplankton. Based on the digitally measured dimensions of 20 random cells per species or size class distinguished (AxioVision 4.9 and AxioCam, Carl Zeiss Microscopy GmbH), biovolumes of MZP were calculated using geometric proxies by Hillebrand et al. [33]. MZP carbon biomass was estimated from the biovolumes using the conversion factors provided by Putt and Stoecker [34] and Menden-Deuer and Lessard [35] for ciliates and dinoflagellates, respectively.

### 2.3 Sampling and identification of phytoplankton

Samples of small-sized phytoplankton (<8 μm) were measured every 2^nd^ day with an Accuri C6 flow cytometer (BD Biosciences) within three hours after mesocosm sampling was completed. Another 250 mL of mesocosm water was fixed with acidic Lugol’s iodine (1% final concentration). Large phytoplankton cells (>8 μm) were counted on ten sampling days around the start of the experiment, the phytoplankton bloom peaks and the end. Counts were done on 50 mL concentrated sample water with an inverted microscope (Zeiss Axiovert 100) after Utermöhl [29]. The cells were counted either on half or total area of the chamber at 100-fold magnification or on 2 to 4 stripes at 200 or 400-fold magnification. Plankton were identified following Tomas et al. [36], Hoppenrath et al. [37], and Kraberg et al. [32].

### 2.4 Data analysis

Diversity (H', log_e_) was calculated after Shannon and Weaver [38] on a sample day basis. The bloom timing (D_max_) was defined as the experimental day when phytoplankton abundance or MZP biomass reached its peak in each mesocosm (max.). Net growth rates μ were calculated using an exponential growth model [39]:

$$\mu =\frac{1}{t}\ln\frac{P_{24}}{P_{0}}$$ (1)

where *P*_*0*_ and *P*_*24*_ are the plankton concentrations on the first day of finding and on D_max_ and *t* is the time in between these days.

D_max_, maximum and net growth rate data were first tested for normality and homogeneity using a Shapiro-Wilk test and Levene's Test for Homogeneity of Variance and log transformed if necessary. For analysing the effects of *p*CO_2_ on these variables, ANOVAs were performed. Tukey’s HSD was used as post-hoc test. To test for significant effects of *p*CO_2_ on MZP and phytoplankton biomass, abundance and species diversity over time, a Generalized Additive Mixed Model (GAMM) was applied. “Mesocosm” was added as random effect to test if there was an effect of the position of the mesocosms on the parameters. Biomass, abundance and diversity data were log transformed if it improved the outcome of the GAMM as indicated by the R^2^ value. R Studio was used for all analyses with the additional packages *mgcv*, *vegan* and *car* (Version 0.95.265, RStudio, Inc.).

### 2.5 Dilution experiment

#### Setup

In order to further investigate the impact of microzooplankton grazing on phytoplankton, a dilution experiment after Landry and Hassett [39] was conducted. The experiment took place on day 34 during the 1^st^ phytoplankton bloom peak to ensure high phytoplankton densities in the samples. By releasing the MZP from copepod grazing pressure, indirect effects of CO_2_ on MZP based on changes in phytoplankton abundance or composition are more likely to become visible. Additionally, this grazing setup allows the determination of the natural taxon-specific phytoplankton growth rates despite of a separation of phytoplankton and micrograzers not being possible due to their similar size.

15 L mesocosm water was sampled with an integrating water sampler from 6 out of 10 mesocosms selected for the experiment. To exclude mesozooplankton, the mesocosm water was pre-screened with a 200 μm mesh. For setting up the dilutions, filtered mesocosm water was obtained by using 0.45/0.2 μm sterile inline membrane filters (Sartobran® P 300, Sartorius AG). Three dilutions of 10, 25 and 50% as well as a setup with 100% undiluted mesocosm water were prepared in carboys. To prevent a bias due to nutrient limitation, sterile filtered nutrient solutions (F/2 medium after [40], reduced by half) were added to the carboys; vitamin solution, trace metal solution, NaNO_3_, Na_2_HPO_4_ (1 mL L^-1^) and Na_2_SiF_6_ (2 mL L^-1^).

In triplicates, the dilutions were gently transferred into 0.5 L polycarbonate incubation bottles to avoid damaging the plankton. Three additional incubation bottles of 100% undiluted water were set up per mesocosm with the addition of five adult copepods (*Pseudocalanus acuspes*) collected from the regular mesozooplankton net tow the day before to analyze mesozooplankton grazing. Animals were picked under a stereomicroscope and were kept in 1 L bottles filled with filtered sea water in the climate room overnight prior to addition to the incubation bottles. Another three incubation bottles per mesocosm were set up without the addition of nutrients to serve as control. Initial samples for MZP and phytoplankton starting densities were obtained from the carboys, transferred into brown glass bottles, and fixed with acid Lugol’s solution. Incubation bottles were placed on a plankton wheel turning at low speed (~1.1 rpm) at ambient temperature (5°C during this experiment) and light conditions in a climate controlled room. After 24 h, samples were taken from every incubation bottle.

MZP was analyzed as described in the previous section. For phytoplankton counts, in principle the same method was used as previously described but with a sample volume between 10 and 50 mL depending on the phytoplankton abundance. At least 400 cells per abundant taxon were counted in tracks of the sedimentation slide using a Zeiss Axiovert 135 inverted microscope. Phytoplankton was identified after Tomas et al. [36] and Kraberg et al. [32] and otherwise assigned to a size class. For the estimation of phytoplankton biovolume 20 pictures per species or size class distinguished were taken (AxioVision 4.9 and AxioCam, Carl Zeiss Microscopy GmbH) and digitally measured with ImageJ (Version 1.49). Phytoplankton biovolumes were calculated from the measurements according to Hillebrand et al. [33] and converted to carbon biomass [34, 35].

From the values obtained from the initial 100% sample, starting values for the diluted samples were calculated according to their dilution factor. Phytoplankton net growth per day μ was calculated using an exponential growth model as described in Eq (1). The actual phytoplankton growth rate *k* and phytoplankton grazing mortality *m* were obtained from a linear regression of the dilution factor α against the phytoplankton growth μ_*α*_ where *k* is the intercept with the y-axis and *m* is the slope of the regression [39, 41]:

$$\mu_{\alpha }=k+m\;\alpha$$ (2)

The MZP grazing rate *g* is the negative phytoplankton mortality. All negative grazing rates were set to zero. To calculate the instantaneous (natural) phytoplankton growth rate μ_*0*_, grazing mortality *m* was added to the net growth rate μ obtained from the controls grown without the addition of nutrients. All data were first tested for normality and homogeneity with a Shapiro-Wilk test and a Levene’s Test for Homogeneity of Variance. Effects of CO_2_ and copepod addition on phytoplankton growth rate *k*, phytoplankton grazing mortality *m* and instantaneous phytoplankton growth rate μ_*0*_ were tested using ANOVAs. Tukey's HSD test was used as post hoc test.

### 2.6 Community grazing experiment

#### Setup

In addition to the dilution experiment, community grazing experiments were performed twice during the mesocosm study. While they do not allow for determination of natural phytoplankton growth rates, phytoplankton net growth rates μ including the MZP grazing impact can be obtained. Moreover, they allow the calculation of MZP growth rates. The time points chosen were day 37 and 53, after the 1^st^ and the 2^nd^ phytoplankton bloom peaks.

From each mesocosm, 5 L of seawater was sampled with an integrating water sampler. Two incubation bottles were set up per mesocosm, one containing unfiltered mesocosm water including mesozooplankton grazers (+G treatment) while the other was filled with pre-screened water (100 μm mesh size) to exclude them (-G treatment). It has to be noted that use of integrated water samplers led to an underestimation of the mesozooplankton grazing impact as copepods were partly able to escape from the sampler. Control of the copepod abundances revealed an abundance reduction by half compared to the mesocosms. Nutrients were not added to the incubation bottles. 250 mL sample of unfiltered and of pre-screened mesocosm water was transferred to brown glass bottles and fixed with acid Lugol’s solution at the beginning of each experiment. Samples from every incubation bottle were taken after 24 h of incubation at ambient conditions using a plankton wheel. MZP and phytoplankton were counted microscopically as described for the dilution experiment.

Phytoplankton and MZP growth rates were calculated with Eq (1) as previously described. Data were tested for normality and homogeneity and transformed (log x+1) if necessary prior to analyzing the effects of CO_2_ and grazer presence using a Two-Way ANOVA.

## 3 Results

**Fig 1:**
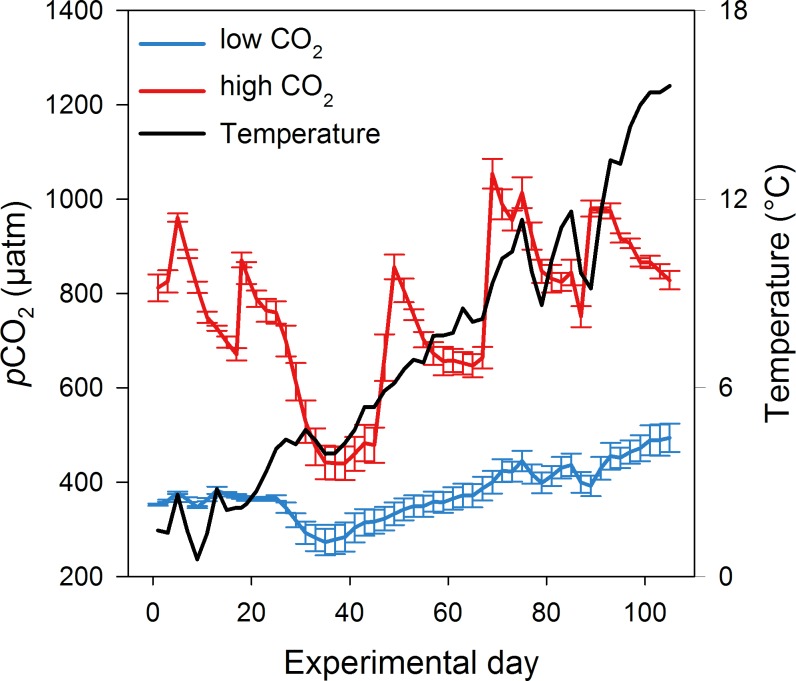
CO_2_ concentration and temperature development. Mean CO_2_ concentrations in the low (blue line) and high CO_2_ treatments (red line) are shown from day 1 to day 105 as well as mean temperature (black line). Error bars indicate the standard deviation.

The experiment ran from 7 March (day -2) until 26 June (day 111). MZP sampling took place from 10 March until 20 June. Within this time, temperature increased from 1.5°C (±0.06) to 15.4°C (±0) (Fig 1). Average *p*CO_2_ was 383 μatm (±100.46) in the low and 739 μatm (±167.11) in the high CO_2_ treatments. Despite of CO_2_ fluctuations in the mesocosms due to outgassing and subsequent addition of CO_2_-enriched water, the treatments did not overlap at any time point.

### 3.1 Phytoplankton succession and community composition

**Fig 2:**
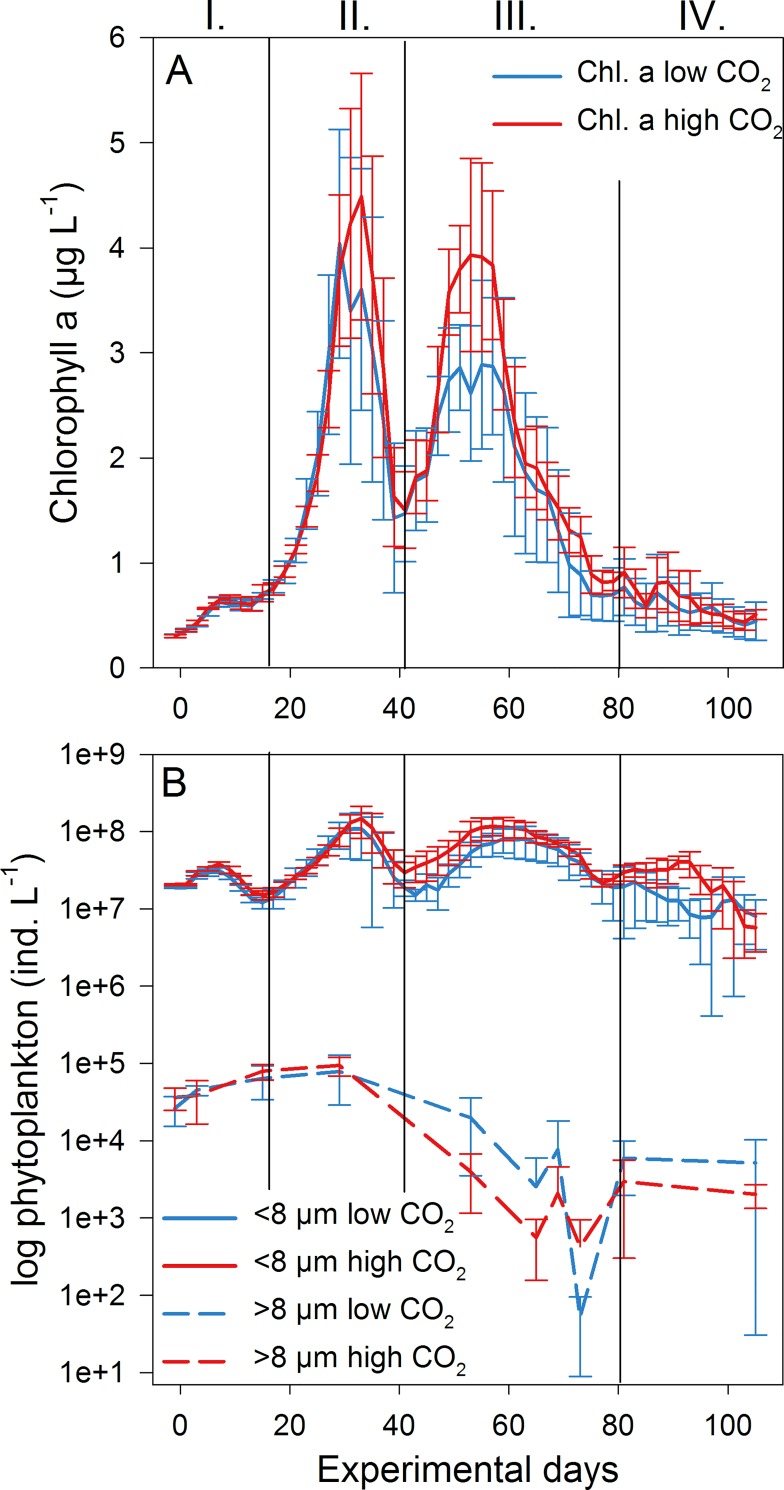
Phytoplankton succession. (A) Chlorophyll *a* concentrations from HPLC analysis in μg L^-1^ at low (blue) and high CO_2_ (red). Error bars represent the standard deviation. Vertical black lines and Latin numbers indicate the experimental phases (I-IV). (B) Phytoplankton abundances in log ind. L^-1^ in the low and high CO_2_ treatments for pico- and nanophytoplankton (<8 μm, solid lines) as well as large phytoplankton (>8 μm, dashed lines).

Starting conditions on 10 March 2013 were Chlorophyll *a* (in the following: Chl *a*) concentrations of 0.363 (±0.014) and 0.357 (±0.013) μg L^-1^ in the low and high CO_2_ mesocosms (Fig 2A). Based on the Chl *a* development, the experiment was divided in four phases: pre-bloom (until day 16), 1^st^ phytoplankton bloom (day 17–40), 2^nd^ phytoplankton bloom (day 41–79) and post-bloom phase (from day 80 on).

Total phytoplankton abundances at the beginning of the experiment (day -1) were at 1.89x10^7^ (±5.06x10^5^) and 2.06x10^7^ (±5.33 x10^5^) cells L^-1^ in the low and high CO_2_ treatments (Fig 2B). During the 1^st^ phytoplankton bloom, the large phytoplankton size class (>8 μm) reached up to 7.91x10^4^ and 9.43x10^4^ cells L^-1^ in the low and high CO_2_ treatments. Afterwards it decreased to ~4.43x10^3^ cells L^-1^ and did not form another bloom. In contrast, small-sized phytoplankton (<8 μm) had abundances of up to 1.48x10^8^ cells L^-1^ during the 1^st^ and 2^nd^ bloom phase under high CO_2_. In the low CO_2_ treatments, this size class reached lower abundances (p-value = 0.010, Table 1) and peaked at 1.10x10^8^ cells L^-1^ and 8.07x10^7^ cells L^-1^ during the 1^st^ and 2^nd^ bloom phase, respectively. On day 105 at the end of the experiment, total phytoplankton abundance was reduced to ~7.70x10^6^ cells L^-1^ with high deviations between mesocosms.

**Table 1:** Results from the GAMM analysis.

Variable		t	p-value	
Abundance	log total phytoplankton	-2.626	0.010	*
	log phytoplankton <8 μm	-2.628	0.010	*
	log phytoplankton >8 μm	0.937	0.351	
	log total ciliates	0.542	0.589	
	log ciliates <30 μm	0.704	0.483	
	log ciliates 30–55 μm	-0.020	0.984	
	log ciliates >55 μm	1.128	0.263	
	Total dinoflagellates	-26.490	<0.001	***
	log dinoflagellates <30 μm	-1.353	0.179	
	Dinoflagellates 30–55 μm	-4.009	<0.001	***
	Dinoflagellates >55 μm	0.315	0.754	
Biomass	log total ciliates	0.945	0.347	
	log ciliates <30 μm	0.519	0.605	
	log ciliates 30–55 μm	-0.154	0.878	
	log ciliates >55 μm	1.302	0.197	
	Total dinoflagellates	-1.872	0.064	
	Dinoflagellates <30 μm	-1.840	0.068	
	Dinoflagellates 30–55 μm	-1.473	0.144	
	Dinoflagellates >55 μm	-0.365	0.716	
Diversity index	Phytoplankton >8 μm	-0.234	0.816	
	Ciliates	-0.264	0.792	
	Dinoflagellates	-0.857	0.393	

Results from the GAMM analysis (Generalized Advanced Mixed Model) of the effects of high CO_2_ on phytoplankton and microzooplankton abundance, biomass, and diversity index. Significances are indicated by asterisks.

**Fig 3:**
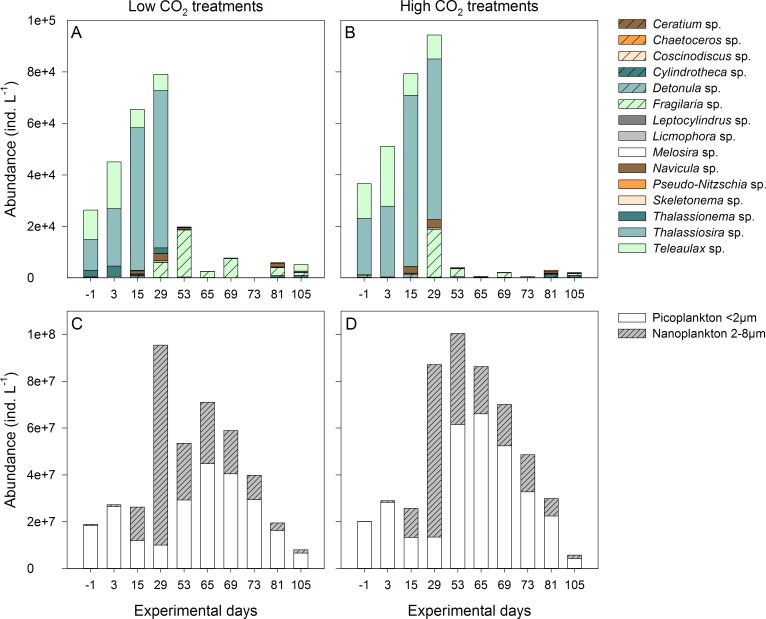
Phytoplankton community composition. Taxonomic composition of the phytoplankton size class >8 μm based on mean abundances from microscopic counts in the (A) low and (B) high CO_2_ treatments in ind. L^-1^ as well as abundances from the size classes <8 μm from flow cytometry in the (C) low and (D) high CO_2_ treatments.

In terms of abundance, pico- and nanophytoplankton of <8 μm contributed up to 99% of the total community. For the size class <8 μm, abundance data from flow cytometry was used as it is more reliable than microscopy for small-sized taxa. Still, microscopic analysis of this size class revealed a dominance of the diatom *Arcocellulus* sp. as well as high abundances of the cryptophyte *Hemiselmis* sp.. Considering phytoplankton of >8 μm which was analyzed by microscopic counts, *Teleaulax* sp. and *Thalassiosira* sp. contributed the main part from the start of the experiment until the 1^st^ bloom (Fig 3). The large phytoplankton community changed considerably after day 29 when both taxa disappeared and the remaining taxa reached very low abundances only, with the exception of *Fragilaria* sp.. Also *Coscinodiscus* sp. increased in numbers after day 29, especially in the low CO_2_ treatments. However, the increase of this large-sized diatom was mostly visible with regard to phytoplankton biomass. Nevertheless, while there was a positive CO_2_ effect on phytoplankton abundance for the size class <8 μm (Fig 2), abundance and species diversity for taxa >8 μm were not affected by the CO_2_ level in the data set we analyzed (Table 1). There was also no effect on phytoplankton abundance maximum or growth rates (Table 2).

**Table 2:** Results for abundance and biomass maxima and growth rates.

Variable		Df	Mean Sq.	F value	p-value	
Abundance max.	log total phytoplankton	1	0.094	1.086	0.328	
	log phytoplankton <8 μm	1	0.094	1.090	0.327	
	log phytoplankton >8 μm	1	0.205	0.562	0.475	
Biomass max.	Total ciliates	1	457.300	1.539	0.250	
	*Strombidium* sp. <40μm	1	0.112	0.324	0.585	
	*Strombidium* sp. >40μm	1	3.999	3.131	0.115	
	*Strobilidium* sp.	1	0.774	0.882	0.375	
	*Tontonia gracillima*	1	3.565	1.779	0.219	
	*Laboea strobila*	1	0.720	0.340	0.578	
	*Lohmaniella oviformis*	1	0.644	0.980	0.351	
	*Myrionecta rubra*	1	0.018	0.238	0.639	
	*Suctoria* sp.	1	0.332	0.440	0.528	
	*Euplotes* sp.	1	0.015	2.180	0.178	
	Total dinoflagellates	1	520.250	5.024	0.055	
	Thecate dinoflagellates <30μm	1	0.042	0.411	0.539	
	Thecate dinoflagellates 30–55μm	1	0.298	0.170	0.691	
	Thecate dinoflagellates >55μm	1	0.589	2.564	0.148	
	Athecate dinoflagellates <30μm	1	1.663	7.137	0.028	*
	Athecate dinoflagellates 30–55μm	1	3.339	4.439	0.068	
	Athecate dinoflagellates >55μm	1	0.256	0.318	0.588	
Growth rate	Total phytoplankton	1	0.304	3.260	0.109	
	Phytoplankton > 8μm	1	0.176	0.427	0.532	
	Total ciliates	1	0.345	1.448	0.263	
	*Strombidium* sp. <40μm	1	0.126	0.359	0.566	
	*Strombidium* sp. >40μm	1	1.727	1.627	0.243	
	*Tontonia gracillima*	1	0.170	0.137	0.724	
	*Laboea strobila*	1	0.041	0.028	0.873	
	*Lohmaniella oviformis*	1	0.226	0.882	0.379	
	Total dinoflagellates	1	1.586	5.807	0.043	*
	Thecate dinoflagellates	1	0.013	0.023	0.883	
	Athecate dinoflagellates <30μμm	1	3.127	12.821	0.007	**
	Athecate dinoflagellates 30–55μm	1	2.400	4.478	0.072	
D_max_	Total ciliates	1	2624.400	21.337	0.002	**
	*Strombidium* sp. <40μm	1	3459.600	15.797	0.004	**
	*Strombidium* sp. >40μm	1	144.400	0.224	0.649	
	*Strobilidium* sp.	1	0.008	0.629	0.451	
	*Tontonia gracillima*	1	0.075	1.084	0.328	
	*Myrionecta rubra*	1	25.600	0.118	0.740	
	Thecate dinoflagellates <30μm	1	3027.600	3.125	0.115	
	Thecate dinoflagellates >55μm	1	48.400	0.074	0.793	

Results from the ANOVAs of the effects of CO_2_ on abundance or biomass maximum (max.), growth rates, and timing of the maximum (D_max_) for phytoplankton, ciliates and dinoflagellates.

Significances are indicated by asterisks.

### 3.2 MZP succession

**Fig 4:**
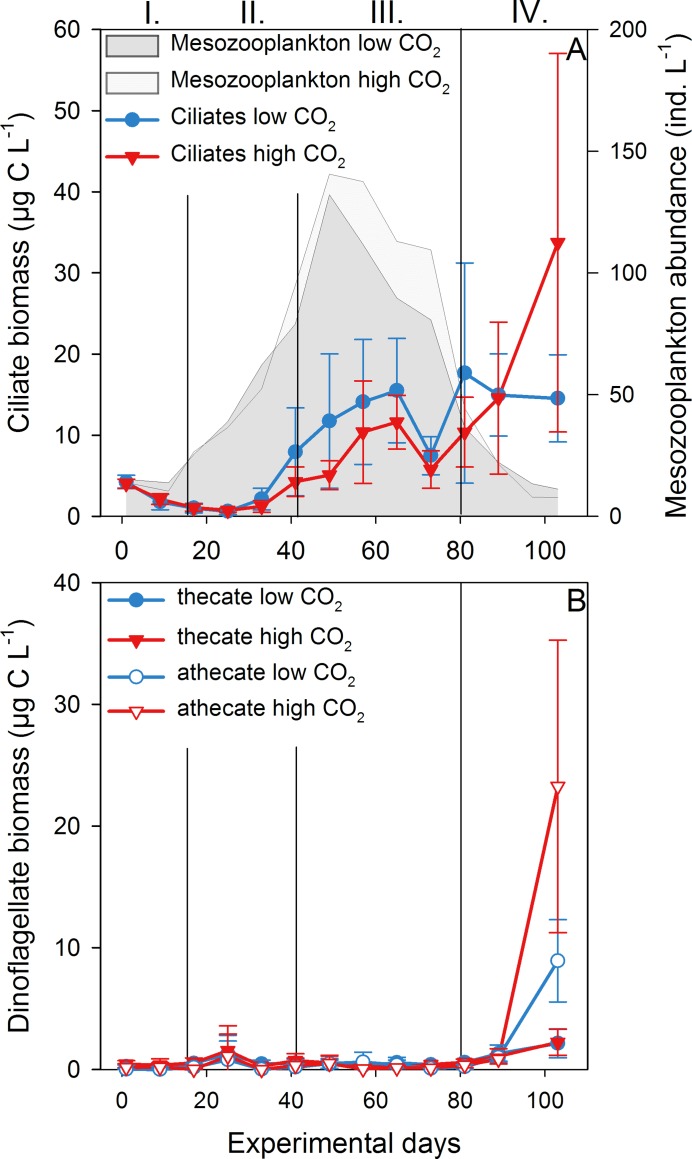
Microzooplankton succession. (A) Ciliate biomass in μg C L^-1^ in the low (blue) and high CO_2_ (red) treatments. Grey fields show total mesozooplankton abundance in ind. L^-1^ at low (dark grey) and high CO_2_ (light grey). Error bars represent the standard deviation and vertical black lines the four experimental phases denoted by the Latin numbers. (B) Biomass of thecate (filled symbols) and athecate dinoflagellates (open symbols) in μg C L^-1^.

Initial ciliate biomass was 4.23 (±0.82) for the low and 4.05 (±0.55) μg C L^-1^ for the high CO_2_ mesocosms (Fig 4A). Ciliate biomasses did not react to the 1^st^ bloom of the phytoplankton; only at the onset of the 2^nd^ bloom on day 40 we observed a first increase in biomass for both treatments. After a decline around day 73, ciliate biomass increased again until day 103, especially in the high CO_2_ treatments. The growth was most pronounced for the high CO_2_ mesocosms MK7 and MK8, reaching 73.18 and 46.74 mg C L^-1^. There was no CO_2_ effect on ciliate biomass, abundance or diversity throughout the experiment (Table 1).

Total dinoflagellate biomass stayed below 1.27 (±1.54) in the low and 1.56 (±2.05) μg C L^-1^ in the high CO_2_ treatments until day 81 (Fig 4B). Afterwards, an increase in athecate dinoflagellates was observed until day 103 where values peaked at 8.92 (±3.38) at low and 23.25 (±12.01) μg C L^-1^ at high CO_2_. Thecate dinoflagellates contributed only ~2 μg C L^-1^ on the last day. While biomass and diversity of dinoflagellates were not affected by the CO_2_ treatment, a positive effect of elevated CO_2_ on the total dinoflagellate abundance was found (p-value < 0.001, Table 1). When size classes were regarded separately, the effect was visible for dinoflagellate abundances from 30–55 μm only (p-value < 0.001).

Analysis of the biomass maxima and growth rates revealed no effect of CO_2_ on total ciliate biomass or ciliate taxa (Table 2). In contrast, the timing of the biomass maximum was significantly later in the high CO_2_ mesocosms for total ciliates (p-value = 0.002) and *Strombidium* sp. <40 μm (p-value = 0.004).

For athecate dinoflagellates <30 μm, a positive effect of CO_2_ on the biomass maximum was observed (p-value = 0.028) while thecate dinoflagellates and total dinoflagellate biomass were not affected. We also found a positive effect of high CO_2_ on the growth rates of total dinoflagellates (p-value = 0.043) and athecate dinoflagellates <30 μm (p-value = 0.007). There was no effect of CO_2_ on the timing of the biomass maxima D_max_.

### 3.3 MZP community composition

**Fig 5:**
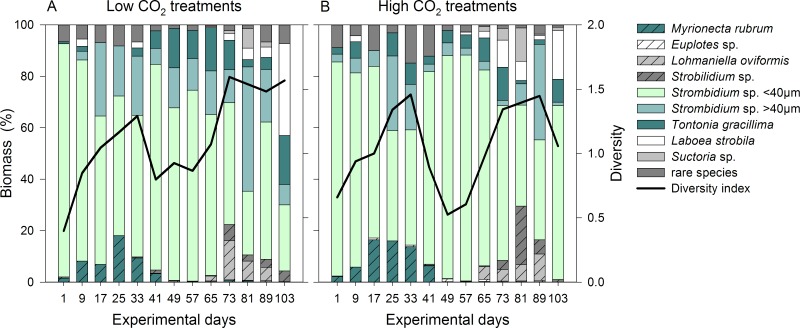
Ciliate community composition and diversity. Taxonomic composition of the ciliate community over the course of the experiment based on mean biomass of the (A) low and (B) high CO_2_ treatments. The black line indicates the species diversity H’.

The ciliate community was dominated by small *Strombidium* sp. <40 μm in both CO_2_ treatments, contributing up to 90% of the total biomass (Fig 5). On day 81 and 89, *Strombidium* sp. >40 μm increased in biomass, providing about half of the total *Strombidium* sp. group. The cyclotrich *Myrionecta rubra* increased in abundance until day 25 where it contributed 18% in the low and 16% in high CO_2_ treatments. It was virtually absent from all mesocosms after day 49. In contrast, the choreotrich *Lohmaniella oviformis* formed a bloom between days 65 and 89 but was not found at other time points. The oligotrich group of *Strobilidium* sp. were present throughout the experiment in small numbers and formed a bloom around day 73, including large *Rimostrombidium* sp.. Large species such as *Tontonia gracillima* and *Laboea strobila* (size class 55–100 μm) were present throughout the experiment. The latter one reached high densities on the last day of the experiment, contributing 36% and 19% in the low and high CO_2_ treatments, respectively. *Suctoria* sp. was found almost exclusively on day 81 in both treatments, in similar densities. The group of rare species included *Balanion comatum*, *Mesodinium pulex*, *Leegardiella* sp., *Tiarina fusus*, *Favella* sp. and *Acineta* sp. in changing proportions at overall low concentrations.

Ciliate diversity was low at the start, increasing until day 33. After a sharp decrease around day 41, caused by the peak of *Strombidium* sp. <40 μm, it increased again afterwards. The diversity of the treatments was not significantly different (Table 1). Although the taxonomic composition of ciliates was very similar in both treatments; standard deviations between single mesocosms were high, especially in the high CO_2_ treatments at the end of the experiment.

**Fig 6:**
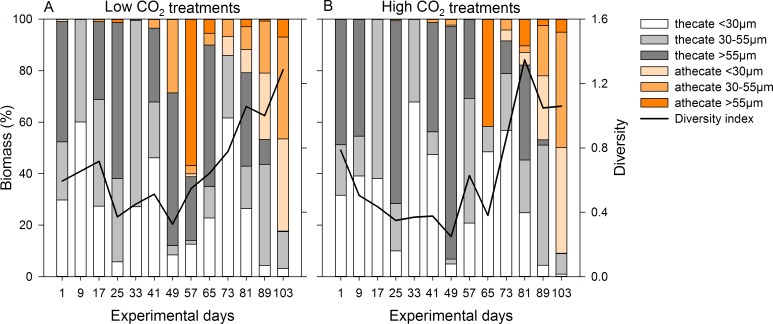
Dinoflagellate community composition and diversity. Size class composition of the thecate (grey bars) and athecate (orange bars) dinoflagellates for the size classes <30 μm, 30–55 μm and >55 μm based on mean biomass of the (A) low and (B) high CO_2_ treatments. Dinoflagellate diversity H’ is indicated by the black line.

During the first half of the experiment, the dinoflagellate community was dominated by thecate dinoflagellates, contributing up to 100% in both CO_2_ treatments (Fig 6). While the contribution of the different size classes varied over time, the main part of the size classes >30 μm was made up by different *Protoperidinium* sp. and *Dinophysis* sp., a mixotrophic taxon. Diversity was around 0.8 at the start and decreased in both treatments until day 49. It increased during the second half of the experiment, starting on day 57 in the low CO_2_ and day 65 in the high CO_2_ treatments. This was due to an increase in athecate dinoflagellates, mainly large *Gyrodinium* sp. (size class >55 μm). After a sharp decrease (low CO_2_: day 65, high CO_2_: day 73), the contribution of athecate dinoflagellates increased again to almost 90% on day 103. In this case, athecate taxa of the size classes <30 μm and 30–55 μm each contributed about half of the community while taxa >55 μm occurred in low numbers only. While this pattern was similar for both treatments, differences in biomass between single mesocosms increased towards the end of the experiment, most notably in the high CO_2_ treatments.

**Fig 7:**
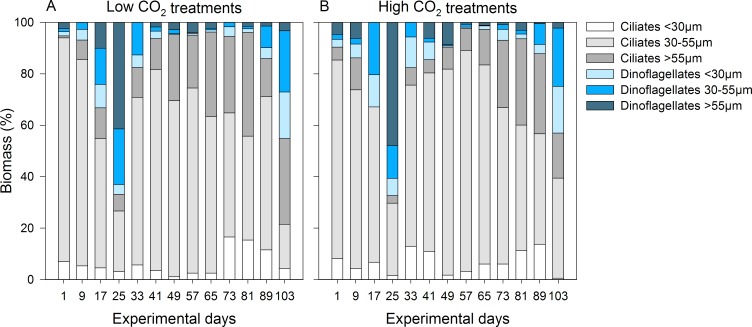
Microzooplankton community composition. Composition of ciliates (grey bars) and dinoflagellates (blue bars) for the size classes <30 μm, 30–55 μm and >55 μm based on mean biomass of the (A) low and (B) high CO_2_ treatments.

Overall, ciliates were the main player of the MZP community in terms of abundance and biomass, showing a similar succession pattern in the two CO_2_ treatments. They contributed 67–98% to the total MZP biomass with the exceptions of day 25 and 103 (Fig 7). On these two occasions, dinoflagellates made up ~66% and ~44%, respectively.

In summary, the MZP community composition or diversity was not affected by CO_2_. MZP was dominated by small *Strombidium* sp., with especially athecate dinoflagellates substantially contributing to the community only during the last days.

### 3.4 Grazing experiments

For the dilution experiment conducted during the 1^st^ phytoplankton bloom peak, growth rates and natural growth rates could be calculated for the four most common phytoplankton groups as well as for total phytoplankton biomass (S1 Table). Phytoplankton growth rates *k* were entirely negative with total phytoplankton, flagellates <5 μm and flagellates >5 μm declining significantly less in the high CO_2_ treatments (p-value < 0.05, Table 3). Likewise, all instantaneous phytoplankton growth rates μ_*0*_ without nutrient addition were negative. However, they declined less under high CO_2_ conditions for total phytoplankton and flagellates <5 μm (p-value < 0.01). Additionally, the actual phytoplankton grazing mortality *m* was higher under high CO_2_ conditions for total phytoplankton and flagellates <5 μm (p-value = 0.002) and close to significant for flagellates >5 μm (p-value = 0.052). We found no effect of CO_2_ on the chlorophyte *Dunaliella* sp. or the diatom *Arcocellulus* sp. for *k*, *m* and μ_*0*_. Furthermore, the presence or absence of copepods had no effect on *k* and μ_*0*_ (data not shown). MZP grazing rates *g* were zero for almost all treatments and could not be further evaluated.

**Table 3:** Results from the dilution experiment.

Variable		Df	Mean Sq	F value	p-value	
*k*	Total phytoplankton	1	0.392	15.587	0.017	*
	Flagellates <5μm	1	0.351	9.854	0.035	*
	Flagellates >5μm	1	1.151	8.254	0.045	*
	*Dunaliella* sp.	1	2.211	6.718	0.061	
	*Arcocellulus* sp.	1	0.049	0.046	0.840	
*m*	Total phytoplankton	1	1.281	37.078	0.004	**
	Flagellates <5μm	1	1.494	57.024	0.002	**
	Flagellates >5μm	1	1.232	7.491	0.052	
	*Dunaliella* sp.	1	1.413	7.278	0.054	
	*Arcocellulus* sp.	1	0.550	0.562	0.495	
μ_*0*_	Total phytoplankton	1	2.333	34.348	0.004	**
	Flagellates <5μm	1	3.091	42.526	0.003	**
	Flagellates >5μm	1	0.865	1.363	0.308	
	*Dunaliella* sp.	1	0.611	1.635	0.270	

Results from the ANOVAs of the effects of CO_2_ on phytoplankton growth rate *k*, phytoplankton grazing mortality *m* and instantaneous phytoplankton growth rate μ_*0*_ without nutrient addition from the dilution experiment.

Significances are indicated by asterisks.

Analysis of the two community grazing experiments revealed no effect of CO_2_ concentration, grazer presence or an interaction of the two factors on net growth rates of total phytoplankton and ciliates (S2 and S3 Tables). There was also no effect on the most common phytoplankton taxa, *Teleaulax* sp., *Arcocellulus* sp., *Paralia sulcata*, flagellates <5 μm and flagellates >5 μm as well as the ciliates *M*. *rubra*, *Strombidium* sp. <40 μm and *Strobilidium* sp. <30 μm. Dinoflagellate growth rates were also not affected in the 1^st^ experiment (Table 4). However, during the 2^nd^ experiment, growth rates of thecate dinoflagellates <30 μm were significantly lower in the high CO_2_ treatment (p-value = 0.012).

**Table 4:** Results from the community grazing experiments.

Experiment	Variable	Factor	Df	Mean Sq	F value	p-value	
1	Total dinoflagellates	CO_2_	1	0.216	0.452	0.511	
		Grazer	1	0.596	1.249	0.280	
		CO_2_ x Grazer	1	0.064	0.134	0.719	
	Thecate dinos <30μm	CO_2_	1	0.530	0.558	0.466	
		Grazer	1	0.014	0.015	0.904	
		CO_2_ x Grazer	1	0.504	0.531	0.477	
	Thecate dinos >30μm	CO_2_	1	0.150	0.123	0.731	
		Grazer	1	1.812	1.490	0.242	
		CO_2_ x Grazer	1	0.038	0.031	0.863	
2	Total dinoflagellates	CO_2_	1	0.001	0.000	0.984	
		Grazer	1	0.738	0.324	0.577	
		CO_2_ x Grazer	1	0.343	0.150	0.703	
	Thecate dinos <30μm	CO_2_	1	6.734	8.659	0.012	*
		Grazer	1	0.401	0.515	0.487	
		CO_2_ x Grazer	1	0.713	0.917	0.357	

Results from the ANOVAs of the effects of CO_2_, grazer presence, and the interaction of the two factors on growth rate of total dinoflagellates and the two size classes of thecate dinoflagellates (dinos) in the community grazing experiments. Significances are indicated by asterisks.

## 4 Discussion

The mesocosms were sustained for more than 100 days by counteracting problems occurring during long-term studies with extensive maintenance work, such as regular cleaning of the in- and outside of the mesocosms to avoid wall growth. While there were fluctuations of *p*CO_2_ for the ambient and the high CO_2_ treatments over time due to photosynthetic carbon fixation and outgassing, there was no overlap between treatments at any time point.

The abundances of MZP in the mesocosms ranged from 4320 to 7489 ind. L^-1^ at the start point which is within the expected range for the Gullmar Fjord area in the beginning of March [42]. MZP abundance stayed comparatively low during the experiment, with the exception of the post-bloom phase for both dinoflagellates and ciliates. Overall, the observed effects of elvated CO_2_ concentrations on the measured MZP parameters were comparatively small and subtle.

### 4.1 Effects on phytoplankton

While some phytoplankton groups like calcifying algae are negatively affected by OA [43, 44], a fertilizing effect on other groups due to the increased availability of carbon has been observed [9, 45, 46]. For example for diatoms, a shift in species composition has been found in different field studies [17] and also changes on genetic level have been observed in laboratory experiments [47]. Comparable to other studies using this mobile mesocosm system, the composition of phytoplankton and the development of abiotic factors such as light and temperature during a spring-bloom situation mimic the natural situation to a high degree. While a 1^st^ bloom peak in the fjord was reached on day 27 with Chl *a* concentrations of 3.52 μg L^-1^, the mesocosm bloom peaked on day 29 in the low and day 33 in the high CO_2_ mesocosms at 4.04 and 4.49 μg L^-1^, respectively.

According to the GAMM analysis, Chl *a* concentrations were significantly higher at high CO_2_ even though the effect seemed to be most pronounced in phase III around the 2^nd^ phytoplankton bloom peak (Fig 2A). We found no effect of high CO_2_ on the abundance of phytoplankton >8 μm. Nonetheless, considering abundances, large sized phytoplankton played only a minor role during both phytoplankton bloom phases. Based on previous studies from other regions, a positive CO_2_ effect on pico- and nanoeukaryotes is more likely to occur [18, 19]. In fact, a positive effect of a high CO_2_ level on picophytoplankton was observed in this experiment [28] which could explain the higher Chl *a* concentrations in these treatments to some extent.

### 4.2 Effects on MZP community composition

A direct effect of a lowered pH on MZP has been shown for areas such as the Baltic Sea or the North Atlantic [11, 12], visible e.g. in the inhibition of growth [10]. However, these effects were only shown for extreme pH values that are unlikely to occur in the near future [10]. Results from a laboratory study applying more realistic OA scenarios on a single MZP species showed no direct effect [13]. In support of that, most mesocosm studies with a CO_2_ level expected for the end of the 21^st^ century also show no or only subtle effects on the MZP community composition and diversity [15, 23, 24]. This can be partly attributed to the high tolerance of coastal communities to frequently occurring habitat pH fluctuations [48, 49]. In general, open ocean communities are considered to be more susceptible to OA as they do not experience these fluctuations, still Rose et al. [14] reported no direct effects of an elevated CO_2_ level on the MZP community in a study in the open Atlantic Ocean.

The aforementioned mesocosm studies lasted 14 to 41 days. Nonetheless, even the longer runtime of 113 days in our study did not result in an effect of the applied CO_2_ level on the MZP community composition and diversity. Thus, hypothesis (1) stating that an elevated CO_2_ level will not directly affect MZP communities due to their high CO_2_ tolerance could not be rejected. In fact, there was an almost parallel development of the composition over time in the two treatments, both for ciliates and heterotrophic dinoflagellates.

### 4.3 Effects on MZP biomass and growth rates

For autotrophic phytoplankton, it has already been shown that high CO_2_ can have an either positive or negative direct impact, depending on the plankton group in focus [16, 20]. In contrast, indirect effects are considered to be more important for heterotrophic or mixotrophic zooplankton than direct ones, such as changes in phytoplankton availability or food quality [14, 50, 51].

Generally, effects of CO_2_ are likely to be more intense in a nutrient-deplete system than in a nutrient-replete one [18, 28, 52] even though this is not always the case, as e.g. in the Arctic Ocean [53]. Nutrient concentrations in the mesocosms were high in the beginning, caused by entrapping nutrient-rich deep water in the mesocosms which is distributed through the whole water column due to wind-induced mixing during wintertime [54]. Nutrient depletion occurred already during the 1^st^ phytoplankton bloom/phase II resulting in the 2^nd^ phytoplankton bloom/phase III being nutrient-deplete and nutrients such as NO_3_^-^/NO_2_^-^, Si(OH)_4_ and PO_4_^3-^ being at concentrations close to or below detection limit [28]. In conclusion, nutrients must have been provided by remineralization to support the observed 2^nd^ bloom, but were immediately used up and thus did not accumulate in the nutrient pool.

While community composition of large phytoplankton was not affected by CO_2_ in the data set we analyzed, positive effects on picoeukaryotes were observed [28]. Additionally, we found a positive effect of high CO_2_ on the abundances of heterotrophic dinoflagellates over time. Total biomass, however, was not affected. This is in agreement with biomass maximum and growth rates of small athecate dinoflagellates <30 μm being higher at elevated CO_2_. The dominance of small athecate dinoflagellates in the community was most likely also the reason why a positive CO_2_ effect on the total dinoflagellate growth rate was found despite of the other size classes not being affected by CO_2_ (Fig 7). Thus, not only phytoplankton but also smaller size classes of microzooplankton seemed to benefit from an elevated CO_2_ level.

A high contribution of athecate dinoflagellates to the total MZP community during the bloom or in the post-bloom phase as in our case has been described for several coastal areas [55]. In contrast, total ciliate biomass was lower under high CO_2_ conditions during the 2^nd^ bloom, even though this effect was not significant. Overall, abundances of large phytoplankton and copepods (Algueró-Muñiz, unpublished data) were not significantly affected by the CO_2_ level in this phase. Still, abundance of pico- and nanophytoplankton was higher at high CO_2_ so the negative effect on MZP was probably caused by additional factors.

The dilution experiment conducted right after the 1^st^ bloom peak took place at a time when MZP abundances were still low. Considering that the phytoplankton community composition was not different from the regular samples from the mesocosms, we can assume that there was no loss of any groups from handling the water samples. The overall decline of phytoplankton was most probably caused by senescence as nutrient addition prevented limitation and the MZP grazing impact was low due to low MZP abundances.

Nonetheless, there was an indication of a higher phytoplankton biomass at high CO_2_ in the dilution experiment as we observed instantaneous phytoplankton growth rates μ_*0*_ declining less in the high CO_2_ treatments. As μ_*0*_ is calculated without the grazing impact, the results suggest that phytoplankton was indeed growing better under high CO_2_, especially small flagellates. This would also fit to Chl *a* maximum of the 1^st^ phytoplankton peak being higher at high CO_2_ and has been described by other authors [16–19]. We also observed an overall higher phytoplankton grazing mortality *m* in the high CO_2_ treatments pointing at a higher MZP abundance at high CO_2_ even though actual grazing rates could not be calculated. This is mirrored in the phytoplankton growth rates *k* declining more at high CO_2_ due to the MZP grazing impact.

Although the two community grazing experiments took place during the 1^st^ and 2^nd^ bloom peak, thus consisting of two somewhat different phytoplankton communities, MZP communities and MZP biomass, the result was the same for both phytoplankton and ciliate growth rates which were not affected by CO_2_. The lack of effect of the grazer treatment for the aforementioned parameters was most likely based on the reduced copepod abundances in the incubation bottles in comparison the mesocosms. Contrastingly, there was once more an effect on dinoflagellates during the 2^nd^ bloom, but in this case a negative CO_2_ effect on the growth rates of heterotrophic thecate dinoflagellates of the size class <30 μm. In addition, we once more found an indication for higher phytoplankton abundances at high CO_2_ in the community grazing experiments which could potentially lead to an increase in MZP biomass. An increase in MZP biomass was indeed what we observed in the mesocosms on day 103 when grazing pressure by copepods had all but disappeared.

We hypothesized that (2) an increase in phytoplankton biomass at high CO_2_ conditions due to positive effects on photosynthesis will lead to enhanced MZP biomass and grazing rates. While grazing rates could not be calculated, hypothesis (2) was confirmed with regard to biomass of dinoflagellates of the size class 30–55 μm as we did observe effects of an elevated CO_2_ level, even though most phytoplankton groups were not affected. However, the hypothesis was rejected for ciliates as this group showed no response except for a delayed bloom peak under high CO_2_.

### 4.4 Food web effects

While an increase in phytoplankton has the potential to positively influence MZP, the effect might be masked by grazing pressure by mesozooplankton as a numerical response of copepods to increasing MZP densities has been described [56]. As mentioned before, copepods are known to be size-selective in their feeding behavior, and while ciliates have the ideal size, phytoplankton cells are often either too small or too large [57]. In our experiment, phytoplankton <8 μm contributed almost 99% of the phytoplankton community during the two blooms in terms of abundance. In general, this size class is considered inedible for most copepod species but represents ideal food items for MZP, especially ciliates [58]. It has already been shown in other experiments that nanoflagellates are selectively grazed upon by ciliates even if other phytoplankton groups are present in sufficient densities [5]. In contrast, dinoflagellates can consume phytoplankton cells larger than their own size and also cannibalistic feeding behavior has been reported [59].

Noticeable was the appearance of large *Coscinodiscus* sp. (>200 μm) during the two phytoplankton blooms, reaching abundances of ~428 ind. L^-1^ during the 2^nd^ bloom and contributing to a large part of the total phytoplankton biomass. However, this large-sized diatom is usually not considered as a copepod food source, even though some copepod species have been reported to feed on them [60]. In conclusion, there was hardly any phytoplankton present in a size class edible for mesozooplankton despite of high phytoplankton abundances during the bloom phases. The low concentration of MZP was therefore most likely caused by intense top-down control by mesozooplankton, thus MZP functioned as a “trophic link” between different levels of the present food web [8].

In addition, grazing pressure could also explain the trend towards higher MZP biomass at high *p*CO_2_ as observed during the post-bloom phase (IV), despite deviations between mesocosms being high. The MZP succession pattern fits to the development of the mesozooplankton population in both CO_2_ treatments (Fig 4A). Starting at low initial abundances, total mesozooplankton increased in numbers reaching on average 136 ind. L^-1^ (±23) on day 49. The dominating mesozooplankton group was copepods, most notably *Pseudocalanus acuspes*. By the end of the 2^nd^ bloom mesozooplankton was reduced to ~40 ind. L^-1^, and continued decreasing even more, thus releasing the MZP from grazing pressure (details on mesozooplankton presented by Algueró-Muñiz, unpublished data). This was the time point when ciliates and dinoflagellates started to increase in the high CO_2_ treatments, despite the decline in phytoplankton densities. While MZP, and especially dinoflagellates, show a variety of feeding mechanisms [55], most taxa are considered as mixotroph and do not necessarily rely on high phytoplankton concentrations alone.

Finally, the occurrence of fish larvae as top-predators of the system had the potential to reduce both copepod and MZP densities. The herring larvae which were released into the mesocosms on day 63 could explain the drop in biomass observed for ciliates on day 73 (Fig 4A) as early stage larval fish are known to feed on MZP [61]. Around day 71, the larvae should have been at an age when they switch from yolk-sack stage to feeding on nauplii and large MZP (>55 μm) [62]. Consequently, large ciliates increased in abundance again afterwards, at the point when the fish larvae started feeding on larger food items such as copepods thus releasing the MZP from grazing pressure. The effect was not visible for large dinoflagellates, probably due to their overall low numbers at this time point.

Apart from grazing pressure by copepods, an explanation for the low MZP biomass at the beginning of the experiment and the lack of response to the 1^st^ phytoplankton bloom (Fig 4A) could have been the low temperatures. In contrast to phytoplankton, which is in large parts light-dependent due to photosynthesis, MZP shows a temperature-dependence due to the biochemical processes of its metabolism [63]. Therefore, a relationship between an increase in temperature and an increase in production has been observed [64–66]. At the beginning of this experiment, sea surface temperatures were ~1°C and during the 1^st^ phytoplankton bloom phase ~5°C (Fig 1). The low temperature seemed to prevent MZP from growth as no biomass increase was observed in response to increases in phytoplankton standing stock. Only during the 2^nd^ phytoplankton bloom phase when temperatures reached up to 10°C, a biomass peak in MZP was observed.

Hypothesis (3) predicted that small sized phytoplankton will profit from high CO_2_ levels which is in favor of MZP, but not mesozooplankton. Overall, it was accepted as an alteration of the phytoplankton community was observed in favor of small-sized phytoplankton. Moreover, as MZP most likely made use of the increase in small phytoplankton while simultaneously being a preferred food item for copepods, the hypothesized increased grazing pressure on MZP due to high CO_2_ was observed.

### 4.5 Conclusion

Complex near-natural systems like the one used in this mesocosms study are associated with a higher buffering capacity towards the effects of OA in comparison with lab studies using simplified food webs [24]. Nevertheless, while we found a high tolerance of most MZP groups to a realistic acidification scenario, we observed effects on both phytoplankton and MZP. While large phytoplankton species remained unaffected by high CO_2_, abundances of small taxa and Chl *a* concentrations were positively affected. We observed no effects on ciliates apart from a delayed bloom peak under high CO_2_. There was, however, a positive effect of CO_2_ on heterotrophic dinoflagellate abundances as well as the biomass maximum and growth rate of athecate dinoflagellates (<30 μm).

This highlights the importance of long-term studies lasting for a complete succession period to follow e.g. an entire build-up and decline during bloom periods in spring. Previous mesocosm studies of comparable size from the Baltic Sea, the North Sea and the Arctic considered only shorter time spans. This might have masked effects of high CO_2_ which are visible only under long-term exposure [15, 23, 24, 67]. Based on the results, MZP communities from coastal regions comparable to the study site are not expected to be strongly affected by end-of-century CO_2_ levels.

## Supporting Information

S1 Table Results from the dilution experiment. Mean values and standard error (Std.error) of the phytoplankton growth rate *k*, instantaneous (natural) phytoplankton growth rate μ_*0*_, phytoplankton mortality *m* and microzooplankton grazing rate *g* are shown for the different phytoplankton groups distinguished in the dilution experiment.
(DOCX)

S2 Table Results from the community grazing experiments. Mean values and standard error (Std.error) of the net growth rates calculated for the most abundant groups of phytoplankton, ciliates and dinoflagellates (dinos) in experiment (Exp.) 1 and 2. The four treatments used were low CO_2_ without grazer (Low -G), low CO_2_ with grazer (Low +G), high CO_2_ without grazer (High -G), and high CO_2_ with grazer (High +G).
(DOCX)

S3 Table Results from the analysis of the community grazing experiments. Results from the ANOVAs from the two community grazing experiments. Effects of CO_2_, grazer presence (Grazer), and the interaction of the two factors on growth rate of total phytoplankton and ciliates as well as the most common taxa of the two groups are shown. Transformations are indicated.
(DOCX)
