# Supplementary material for: Low CO2 Sensitivity of Microzooplankton Communities in the Gullmar Fjord, Skagerrak: Evidence from a Long-Term Mesocosm Study
Source: PLoS One. 2016 Nov 28;11(11):e0165800. doi: 10.1371/journal.pone.0165800 (PMC5125589; doi:10.1371/journal.pone.0165800)
Supplement: S1 Table — Mean values and standard error (Std.error) of the phytoplankton growth rate k, instantaneous (natural) phytoplankton growth rate μ0, phytoplankton mortality m and microzooplankton grazing rate g are shown for the different phytoplankton groups distinguished in the dilution experiment. (DOCX) [file pone.0165800.s001.docx]

**S1 Table: Results from the dilution experiment.**

| Variable | Phytoplankton group | low CO_2_ | Std. error | high CO_2_ | Std. error |
| --- | --- | --- | --- | --- | --- |
| *k* | Total phytoplankton | -1.550 | 0.099 | -2.061 | 0.038 |
|  | Flagellates <5µm | -1.526 | 0.117 | -2.010 | 0.046 |
|  | Flagellates >5µm | -1.510 | 0.184 | -2.386 | 0.168 |
|  | *Dunaliella* sp. | -1.324 | 0.063 | -2.538 | 0.377 |
|  | *Arcocellulus* sp. | -2.806 | 0.658 | -2.986 | 0.193 |
| *µ0* | Total phytoplankton | -1.746 | 0.114 | -0.499 | 0.131 |
|  | Flagellates <5µm | -1.893 | 0.133 | -0.457 | 0.121 |
|  | Flagellates >5µm | -1.339 | 0.526 | -0.580 | 0.077 |
|  | *Dunaliella* sp. | -0.424 | 0.262 | 0.214 | 0.312 |
|  | *Arcocellulus* sp. | -3.230 | 1.575 | -1.819 | 0.235 |
| *m* | Total phytoplankton | -0.127 | 0.104 | 0.797 | 0.067 |
|  | Flagellates <5µm | -0.206 | 0.086 | 0.792 | 0.065 |
|  | Flagellates >5µm | 0.036 | 0.266 | 0.942 | 0.049 |
|  | *Dunaliella* sp. | 0.404 | 0.159 | 1.374 | 0.247 |
|  | *Arcocellulus* sp. | 0.009 | 0.652 | 0.615 | 0.099 |
| *g* | Total phytoplankton | 0.127 | 0.104 | 0 |  |
|  | Flagellates <5µm | 0.206 | 0.086 | 0 |  |
|  | Flagellates >5µm | 0 |  | 0 |  |
|  | *Dunaliella* sp. | 0 |  | 0 |  |
|  | *Arcocellulus* sp. | 0 |  | 0 |  |

Mean values and standard error (Std.error) of the phytoplankton growth rate *k*, instantaneous phytoplankton growth rate *µ0*, phytoplankton mortality *m* and microzooplankton grazing rate *g* are shown for the different phytoplankton groups distinguished in the dilution experiment.
