# Supplementary material for: Low CO2 Sensitivity of Microzooplankton Communities in the Gullmar Fjord, Skagerrak: Evidence from a Long-Term Mesocosm Study
Source: PLoS One. 2016 Nov 28;11(11):e0165800. doi: 10.1371/journal.pone.0165800 (PMC5125589; doi:10.1371/journal.pone.0165800)
Supplement: S2 Table — Mean values and standard error (Std.error) of the net growth rates calculated for the most abundant groups of phytoplankton, ciliates and dinoflagellates (dinos) in experiment (Exp.) 1 and 2. The four treatments used were low CO2 without grazer (Low -G), low CO2 with grazer (Low +G), high CO2 without grazer (High -G), and high CO2 with grazer (High +G). (DOCX) [file pone.0165800.s002.docx]

**S2 Table: Results from the community grazing experiment.**

| Exp. | Variable | Low  -G | Std.  error | Low  +G | Std.  error | High  -G | Std.  error | High  +G | Std.  error |
| --- | --- | --- | --- | --- | --- | --- | --- | --- | --- |
| 1 | Total phytoplankton | -0.586 | 0.156 | -0.663 | 0.428 | -0.675 | 0.214 | -0.430 | 0.318 |
|  | Flagellates <5 µm | -0.712 | 0.152 | -0.738 | 0.464 | -0.794 | 0.202 | -0.665 | 0.585 |
|  | Flagellates >5 µm | -0.388 | 0.375 | -0.978 | 0.336 | -0.387 | 0.391 | -0.583 | 0.146 |
|  | *Teleaulax* sp. | 0.015 | 0.466 | 0.107 | 0.478 | -0.367 | 0.558 | 0.327 | 0.021 |
|  | *Paralia sulcata* | -0.148 | 0.380 | 0.164 | 0.724 | -0.397 | 0.616 | 0.693 | 0.129 |
|  | Total ciliates | -0.047 | 0.138 | 0.121 | 0.137 | 0.331 | 0.266 | -0.205 | 0.126 |
|  | Total dinoflagellates | 0.298 | 0.357 | 0.531 | 0.194 | -0.022 | 0.113 | 0.436 | 0.358 |
|  | *Myrionecta rubra* | -0.150 | 0.344 | 0.732 | 0.355 | 0.010 | 0.425 | -0.114 | 0.086 |
|  | *Strobilidium* sp. <30µm | 0.253 | 0.410 | 0.473 | 0.486 | -0.669 | 0.436 | -0.623 | 0.488 |
|  | *Strombidium* sp. <30µm | 0.030 | 0.106 | 0.061 | 0.141 | 0.394 | 0.364 | -0.238 | 0.145 |
|  | Thecate dinos <30µm | 0.308 | 0.370 | 0.679 | 0.227 | 0.300 | 0.588 | 0.036 | 0.271 |
|  | Thecate dinos >30µm | -0.207 | 0.382 | 0.530 | 0.500 | -0.298 | 0.396 | 0.255 | 0.521 |
| 2 | Total phytoplankton | 0.120 | 0.182 | -0.108 | 0.074 | 0.057 | 0.078 | -0.236 | 0.187 |
|  | Flagellates <5 µm | 0.151 | 0.141 | 0.250 | 0.102 | 0.011 | 0.095 | 0.062 | 0.074 |
|  | Flagellates >5 µm | 0.208 | 0.347 | -0.027 | 0.210 | 0.303 | 0.449 | -0.284 | 0.430 |
|  | *Arcocellulus* sp. | -0.074 | 0.599 | -0.565 | 0.547 | -0.418 | 0.472 | 0.109 | 0.420 |
|  | Total ciliates | 0.066 | 0.116 | 0.105 | 0.091 | 0.658 | 0.094 | 0.160 | 0.215 |
|  | Total dinoflagellates | -0.546 | 0.786 | -0.423 | 0.282 | -0.821 | 0.179 | -0.175 | 0.853 |
|  | *Myrionecta rubra* | 0.224 | 0.204 | 0.100 | 0.317 | 0.246 | 0.264 | 0.385 | 0.475 |
|  | *Strobilidium* sp. <30µm | -0.034 | 0.191 | 0.257 | 0.477 | 0.648 | 0.491 | -0.438 | 0.691 |
|  | *Strombidium* sp. <30µm | 0.042 | 0.112 | 0.097 | 0.132 | 0.671 | 0.098 | 0.067 | 0.276 |
|  | Thecate dinos <30µm | 0.259 | 0.397 | 0.997 | 0.428 | -0.617 | 0.298 | -0.723 | 0.391 |

Mean values and standard error (Std.error) of the net growth rates calculated for the most abundant groups of phytoplankton, ciliates and dinoflagellates (dinos) in experiment (Exp.) 1 and 2. The four treatments used were low CO_2_ without grazer (Low -G), low CO_2_ with grazer (Low +G), high CO_2_ without grazer (High -G), and high CO_2_ with grazer (High +G).
