# Supplementary material for: Low CO2 Sensitivity of Microzooplankton Communities in the Gullmar Fjord, Skagerrak: Evidence from a Long-Term Mesocosm Study
Source: PLoS One. 2016 Nov 28;11(11):e0165800. doi: 10.1371/journal.pone.0165800 (PMC5125589; doi:10.1371/journal.pone.0165800)
Supplement: S3 Table — Results from the ANOVAs from the two community grazing experiments. Effects of CO2, grazer presence (Grazer), and the interaction of the two factors on growth rate of total phytoplankton and ciliates as well as the most common taxa of the two groups are shown. Transformations are indicated. (DOCX) [file pone.0165800.s003.docx]

**S3 Table: Results from the analysis of the community grazing experiments.**

| Experiment | Variable | Factor | Df | Mean Sq | F value | p-value |
| --- | --- | --- | --- | --- | --- | --- |
| 1 | Total phytoplankton | CO_2_ | 1 | 0.026 | 0.047 | 0.831 |
|  |  | Grazer | 1 | 0.035 | 0.064 | 0.804 |
|  |  | CO_2_ x Grazer | 1 | 0.129 | 0.233 | 0.636 |
|  | Flagellates <5µm | CO_2_ | 1 | 0.000 | 0.000 | 0.992 |
|  |  | Grazer | 1 | 0.013 | 0.014 | 0.909 |
|  |  | CO_2_ x Grazer | 1 | 0.030 | 0.030 | 0.864 |
|  | Flagellates >5µm | CO_2_ | 1 | 0.196 | 0.293 | 0.596 |
|  |  | Grazer | 1 | 0.773 | 1.157 | 0.298 |
|  |  | CO_2_ x Grazer | 1 | 0.194 | 0.290 | 0.598 |
|  | *Teleaulax* sp. | CO_2_ | 1 | 0.023 | 0.021 | 0.890 |
|  |  | Grazer | 1 | 0.261 | 0.235 | 0.641 |
|  |  | CO_2_ x Grazer | 1 | 0.236 | 0.212 | 0.658 |
|  | *Paralia sulcata* | CO_2_ | 1 | 0.006 | 0.004 | 0.953 |
|  |  | Grazer | 1 | 1.296 | 0.769 | 0.401 |
|  |  | CO_2_ x Grazer | 1 | 0.485 | 0.288 | 0.603 |
| 1 | Total ciliates log (x+1) | CO_2_ | 1 | 0.002 | 0.009 | 0.925 |
|  |  | Grazer | 1 | 0.124 | 0.669 | 0.425 |
|  |  | CO_2_ x Grazer | 1 | 0.604 | 3.260 | 0.090 |
|  | *Myrionecta rubra* | CO_2_ | 1 | 0.589 | 0.870 | 0.365 |
|  |  | Grazer | 1 | 0.719 | 1.064 | 0.318 |
|  |  | CO_2_ x Grazer | 1 | 1.267 | 1.873 | 0.190 |
|  | *Strobilidium* sp. <30µm | CO_2_ | 1 | 4.940 | 3.835 | 0.069 |
|  |  | Grazer | 1 | 0.078 | 0.060 | 0.809 |
|  |  | CO_2_ x Grazer | 1 | 0.036 | 0.028 | 0.870 |
|  | *Strombidium* sp.<40µm | CO_2_ | 1 | 0.049 | 0.228 | 0.639 |
|  | log (x+1) | Grazer | 1 | 0.410 | 1.906 | 0.186 |
|  |  | CO_2_ x Grazer | 1 | 0.448 | 2.081 | 0.168 |
| 2 | Total phytoplankton | CO_2_ | 1 | 0.045 | 0.365 | 0.554 |
|  |  | Grazer | 1 | 0.340 | 2.730 | 0.118 |
|  |  | CO_2_ x Grazer | 1 | 0.005 | 0.042 | 0.841 |
|  | Flagellates <5µm | CO_2_ | 1 | 0.134 | 1.913 | 0.186 |
|  |  | Grazer | 1 | 0.028 | 0.406 | 0.533 |
|  |  | CO_2_ x Grazer | 1 | 0.003 | 0.041 | 0.843 |
|  | Flagellates >5µm | CO_2_ | 1 | 0.036 | 0.046 | 0.834 |
|  |  | Grazer | 1 | 0.668 | 0.864 | 0.373 |
|  |  | CO_2_ x Grazer | 1 | 0.115 | 0.148 | 0.708 |
|  | *Arcocellulus* sp. | CO_2_ | 1 | 0.199 | 0.155 | 0.702 |
|  |  | Grazer | 1 | 0.001 | 0.001 | 0.978 |
|  |  | CO_2_ x Grazer | 1 | 0.887 | 0.691 | 0.425 |
| 2 | Total ciliates log (x+1) | CO_2_ | 1 | 0.225 | 2.256 | 0.153 |
|  |  | Grazer | 1 | 0.205 | 2.060 | 0.170 |
|  |  | CO_2_ x Grazer | 1 | 0.307 | 3.081 | 0.098 |
|  | *Myrionecta rubra* | CO_2_ | 1 | 0.124 | 0.180 | 0.678 |
|  |  | Grazer | 1 | 0.000 | 0.000 | 0.999 |
|  |  | CO_2_ x Grazer | 1 | 0.081 | 0.119 | 0.735 |
|  | *Strobilidium* sp. <30µm | CO_2_ | 1 | 0.067 | 0.055 | 0.818 |
|  |  | Grazer | 1 | 0.546 | 0.451 | 0.515 |
|  |  | CO_2_ x Grazer | 1 | 1.835 | 1.515 | 0.242 |
|  | *Strombidium* sp.<40µm | CO_2_ | 1 | 0.448 | 2.481 | 0.135 |
|  |  | Grazer | 1 | 0.377 | 2.088 | 0.168 |
|  |  | CO_2_ x Grazer | 1 | 0.542 | 3.000 | 0.103 |

Results from the ANOVAs from the two community grazing experiments. Effects of CO_2_, grazer presence, and the interaction of the two factors on growth rate of total phytoplankton and ciliates as well as the most common taxa of the two groups are shown. Transformations are indicated.
